# High expression ITGA2 affects the expression of MET, PD-L1, CD4 and CD8 with the immune microenvironment in pancreatic cancer patients

**DOI:** 10.3389/fimmu.2023.1209367

**Published:** 2023-10-10

**Authors:** Liquan Jin, Yaoqiang Duan, Xiaoxi Li, Zhenqi Li, Jifu Hu, Hongbo Shi, Ziting Su, Zhe Li, Bilian Du, Yiming Chen, Yunbo Tan

**Affiliations:** ^1^ 1St Department of General Surgery, The First Affiliated Hospital of Dali University, Dali, Yunnan, China; ^2^ Clinical Medical College of Dali University, Dali, Yunnan, China

**Keywords:** pancreatic cancer, ITGA2, EMT, PD-L1, microenvironment

## Abstract

**Purpose:**

Pancreatic cancer is characterized by a grim prognosis and is regarded as one of the most formidable malignancies. Among the genes exhibiting high expression in different tumor tissues, ITGA2 stands out as a promising candidate for cancer therapy. The promotion of cancer in pancreatic cancer is not effective. The objective of this study is to assess the presence of ITGA2, EMT and PD-L1 in pancreatic cancer.

**Experimental design:**

We examined the expression of ITGA2, MET, E-cadherin, PD-L1, CD4, and CD8 proteins in 62 pancreatic cancer tissue samples using multi-tissue immunofluorescence and immunohistochemistry techniques. Functional assays, such as the cell migration assay and transwell assay, were used to determine the biological role of ITGA2 in pancreatic cancer. The relationship of ITGA2,EMT and PD-L1 were examined using Western blot analysis and RT-qPCR assay.

**Results:**

In our study, we observed the expression of ITGA2, E-cadherin, and PD-L1 in both tumor and stroma tissues of pancreatic cancer. Additionally, a positive correlation between ITGA2, E-cadherin, and PD-L1 in the tumor region (r=0.559, P<0.001 and r=0.511, P<0.001), and PD-L1 in the stroma region (r=0.512, P<0.001).The expression levels of ITGA2, CD4, and CD8 were found to be higher in pancreatic cancer tissues compared to adjacent tissues (P < 0.05). Additionally, ITGA2 was negatively correlated with CD4 and CD8 (r = -0.344, P < 0.005 and r = -0.398, P < 0.005).Furthermore, ITGA2, CD4, and CD8 were found to be correlated with the survival time of patients (P < 0.05). Blocking ITGA2 inhibited the proliferation and invasion ability of pancreatic cancer cells significantly, Additionally, sh-ITGA2 can down-regulate the expression of EMT and PD-L1.

**Conclusions:**

We identified a novel mechanism in which ITGA2 plays a crucial role in the regulation of pancreatic cancer growth and invasion. This mechanism involves the upregulation of MET and PD-L1 expression in pancreatic cancer cells. Additionally, we found that increased expression of ITGA2 is associated with a poor prognosis in pancreatic cancer patients. Furthermore, ITGA2 also affects immune regulation in these patients. Therefore, targeting ITGA2 is an effective method to enhance the efficacy of checkpoint immunotherapy and prohibiting tumor growth against pancreatic cancer.

## Introduction

Pancreatic cancer (PC) is a highly lethal tumor with a poor prognosis, ranking as the fourth or fifth most common cause of cancer death in developed countries (1). Unfortunately, the incidence of pancreatic cancer is on the rise, with projections suggesting it will become the second leading cause of cancer deaths by 2020 (2). The incidence of pancreatic cancer is higher in urban areas, with rates 2 to 3 times higher than in rural areas. Unfortunately, the survival time for pancreatic cancer patients has not improved as much as other cancer types. In fact, there has been a slight decrease in the survival time for pancreatic cancer over time. This is likely because most cases of pancreatic cancer are diagnosed at an advanced stage, resulting in a low 5-year survival rate of only 8% (3). Therefore, further studies on the pathogenesis of pancreatic cancer are essential to improve the survival time of patients with pancreatic cancer.

Pancreatic cancer research has increasingly focused on the tumor microenvironment. Recent research has highlighted the crucial role played by the pancreatic tumor microenvironment in the progression of PDAC, with a clear link established between the microenvironment and metastasis. The microenvironment of pancreatic cancer is characterized by two main features: dense fibrous tissue proliferation and extensive immunosuppression (4). These properties can promote the proliferation of pancreatic cancer cells and evade immune surveillance by inhibiting anti-tumor immunity directly or inducing the proliferation and metastasis of immunosuppressive cells and the epithelial-to-mesenchymal transition (EMT) plays a crucial role in the tumor microenvironment of pancreatic cancer. Specifically, EMT is characterized by the loss of adhesion ability between cells during the process of metastasis. EMT plays a crucial role in several pathological processes such as wound healing, tissue fibrosis, and cancer progression (5, 6). It is closely linked to the proliferation of dense fibrous tissue in the microenvironment of pancreatic cancer. Studies have shown that maintenance of the mesenchymal phenotype, as well as inflammation, can enhance EMT, invasiveness, and dissemination of pancreatic epithelial cells, even at the PanIN stage (7). Research has indicated that the co-activation of ZEB1 and YAP1 can trigger the transcriptional regulation of ITGA3 through the binding sites of YAP1/TEAD in both pancreatic cancer cells and tissues. This activation subsequently leads to epithelial-mesenchymal transition (EMT) plasticity and metastasis (8). In typical pancreatic ductal adenocarcinoma (PDA), the tumor microenvironment (TME) often exhibits an immuno-”cold” characteristic. This is characterized by significant infiltration of myeloid cells, but a lack of CD8+ T cells (Teffector) and low expression of activation markers such as GZMB and IFNG. These indicators suggest the absence or dysfunction of adaptive T-cell immunity and resistance to checkpoint blockade (9). Tumor of endogenous and exogenous factors, such as low tumor mutation load, and hinder the T cells launch, transportation, and function of complex immune inhibitory TME, exacerbated by the PDA adaptive immunity (10–12). Therefore, investigating the simultaneous modulation of epithelial-mesenchymal transition (EMT) in the tumor microenvironment of pancreatic cancer and the molecular regulation of immune cell function is crucial for effective pancreatic cancer treatment.

Integrins are heterodimeric transmembrane glycoproteins that provide the main adhesion sites for cells and mediate the adhesion of cells to the surrounding microenvironment Cells, allowing them to mechanically communicate with each other. Properties of dynamics Integrin receptors enable cells to sense and adjust mechanical and chemical signals Starting from the environment and vice versa, force is applied to the surrounding environment (13). The overexpression of ITGA2 has been found to enhance cell proliferation and promote cancer cell invasion through the activation of the PD-L1 and EMT in various cancers (14, 15). Numerous studies have suggested that integrins play a critical role in regulating cell adhesion, invasion, tumor progression and metastasis (16, 17). However, little is known about the prognostic impact of TIGA2,EMT and PD-L1 in PDAC, and there are no studies investigating expression and a possible interrelationship between ITGA2 and MET/PD-L1. Targeting ITGA2 could represent a promising strategy for cancer treatment.

The objective of this study was to examine the association between the expressions of ITGA2, MET, PD-L1, and T cell biomarkers (CD4 and CD8), and the characteristics of PDAC tumors as well as the survival of PDAC patients. Additionally, we aimed to investigate the relationship between ITGA2 and EMT/PD-L1 in pancreatic cancer cells. Our findings suggest that targeting ITGA2 could potentially be an effective approach to improve the effectiveness of checkpoint immunotherapy and inhibit the progression of pancreatic cancer.

## Materials and methods

### Patient population and tissue sampling

This study involved a total of 62 patients diagnosed with pancreatic cancer who underwent resection surgeries with curative intent at the Unit of General Surgery, University - The First Affiliated Hospital of Dali. Of these patients, 6 had high differentiation, 45 had moderate differentiation, and 11 exhibited low differentiation. Ethical approval for this study was granted by the Ethical Committee of the University’s First Affiliated Hospital of Dali. The study was conducted in accordance with the principles outlined in the Helsinki Declaration. Patients were enrolled only after providing informed consent for the use of their biological samples for research purposes. The patient population that underwent surgical resection (as shown in **Table S1**) included 40 males and 22 females aged between 45 and 79 years. The tumor stage was determined using the 7th American Joint Committee on Cancer (AJCC) staging system, while **Table S1** provides details on histological diagnosis, pathological staging, and tumor markers.

### Cells and culture conditions

For this study, Human normal pancreatic ductal epithelial cells(HPDE-C7) and pancreatic cancer cell line PANC-1 and SW1990 were purchased from the American Type Culture Collection (IMMOCELL^®^, PROCELL^®^ and Servicebio^®^, WuHan, China). Cells were maintained in High sugar DMEM medium (Servicebio, WuHan, China) supplemented with 10% heat-inactivated foetal calf serum (PROCELL, WuHan, China) and 1% penicillin/streptomycin (Servicebio, WuHan, China) and grown at 37°CC, at 5% CO2 in a humidified incubator. Cells were tested monthly for mycoplasma contamination using the MycoAlert Mycoplasma Detection kit (Westburg, Leusden, The Netherlands).

### Plasmids transfection

RNA interference The sh-Control and sh-ITGA2s were procured from Sigma-Aldrich. Lipo8000TM (Beyotime, China) and High sugar DMEM media (Servicebio^®^, WuHan, China) were used for the transfection reactions;Lipo8000TM transfection reagent(GENERAL BIOL, China) was used to shRNA plasmids.24 h after transfection, the medium was replaced with fresh DMEM containing 10% FBS. The shRNA sequence information is provided in the **Additional File 1: Table S2**.

### Western blot analysis

To achieve complete cell lysis, 0.020 g of cells were mixed with RIPA lysis buffer (Epizyme Biotech Co., Ltd.) and the resulting mixture was centrifuged at 1,400 x g at 4°C for 20 minutes. After centrifugation, the supernatant was collected. The protein content in the supernatant was quantified using the BCA method (Epizyme Biotech Co., Ltd.), and protein samples were prepared for further analysis. A 10% SDS-PAGE gel was prepared to separate the samples. A total of 100 ng of protein extract was loaded per lane. After electrophoresis, the protein extracts were transferred onto PVDF membranes. The PVDF membranes were then blocked with a blocking reagent (5% skimmed dried milk) for 1 hour at room temperature. The primary antibodies used were Rabbit Monoclonal antibody ITGA2 (1:300, bioss, Beijing, China, No: bs-10132R), Rabbit Monoclonal antibody E-cadherin (1:1000, Cell Signaling Technology, USA, No. 3195S), Rabbit Monoclonal antibody N-cadherin (1:1000, Cell Signaling Technology, USA, No. 13116S), Rabbit Polyclonal antibody Snail1 (1:700, ZEN-BIOSCIENCE, China, No. 340942), and Rabbit Monoclonal antibody PD-L1 (1:700, ZEN-BIOSCIENCE, China, No. R30023). The primary antibodies were added and incubated overnight at 4°C on a shaking table. The TBST membrane was cleaned. After incubating with HRP Goat anti-rabbit antibodies (1:500, Epizyme Biotech, China, no. LF102) for 1 hour at room temperature, the TBST membrane was washed. An ECL kit (Fdbio Science) was used for development. The gray value of each strip was analyzed using ImageJ 1.6 software (National Institutes of Health) with the assistance of a gel imaging system from Bio-Rad Laboratories, Inc.

### Reverse transcription-quantitative PCR

Cells were seeded into 6-well plates at a density of 0.7x103cells/well. The total RNA was isolated according to the manufacturer’s protocols using the RNAqueous micro kit (cat. no. AM 1931; Ambion; Thermo Fisher Scientific, Inc.). For cDNA synthesis, a high-capacity cDNA reverse transcription kit (cat. no. 4368814; Applied Biosystems; Thermo Fisher Scientific, Inc.) was used according to the manufacturer’s protocols. The ABI Prism 7300 sequence detection system (Applied Biosystems; Thermo Fisher Scientific, Inc.) was used to run qPCR for *ITGA2*,*N-cadherin*,*C-cadherin*,*Snail1*,*GAPDH* in accordance with the instructions of the manufacturer using the ChamQ SYBR qPCR Master Mix (cat. no. Q311-02; Vazyme Biotech, Co., Ltd.). Endogenous control was GAPDH; m*ITGA2*: Sense5’-3’ TGAGAACCGAATGGGAGATGTG;antisense5’-3’ CAACATCTATGAGGGAAGGGCAG;m*N-cadherin*: Sense5’-3’GCCACCTACAAAGGCAGAAGAG; antisense5’-3’ CCTCAAATGAAACCGGGCTAT;m*E-cadherin*: Sense5’-3’ ACGCATTGCCACATACACTCTC; antisense5’-3’GCACCTTCCATGACAGACCC;m*Snail1*:Sense5’-3’ GACCCCAATCGGAAGCCTAAC; antisense5’-3’ATGAGCATTGGCAGCGAGG;m*PD-L1*:Sense5’-3’ GCCGAAGTCATCTGGACAAGC; antisense 5’-3’GTGTTGATTCTCAGTGTGCTGGTCA; m*GAPDH*: Sense5’-3’AGGAAGCTTGTCATCAATGGAAATC;antisense5’-3’TGATGACCCTTTTGGCTCCC. The total volume of each reaction was 20 µl. The PCR thermocycling conditions were as follows: 95°C for 5 min, with 40 cycles of 95°C for 10 sec, 58°C for 30 sec and 70°C for 10 sec. In a 20-µl reaction volume, all samples were run in triplicate and the results were calculated as threshold cycle (CT) values. The expression levels were calculated using delta CT. The data were determined as the mean of three independent measurements and the relative mRNA expression level was determined with the 2-ΔΔCq model (18).

### Cell migration

A MAKER pen was utilized to draw horizontal lines on the back of the six-well plate, using a ruler to ensure intervals of 0.5cm-1.0cm between each line. At least three horizontal lines were drawn per well. Next, 50×104 cells were added to each well (40×10^4^ cells can be used for SW1990 cells). It is important to ensure even distribution of the cells. After 24 hours, when the cells have adhered to the plate, a sterilized 200μl spear tip was used to create a perpendicular line intersecting the previously marked horizontal line. The cells within the scratch were removed, the culture medium was aspirated, and PBS2 was added to the well to eliminate any remaining cells. Subsequently, serum-free or low-serum medium was added to the well to observe cell invasion at 6 hours, 12 hours, 24 hours, 36 hours and 48 hours.

### Cell invasion assay

The transwell assay was employed to assess cell invasion. 24-well Corning Costar inserts with 8-μm pores, precoated with Matrigel (BD, United States; diluted 1:8), were employed. The inserts were incubated for 6 hours in an incubator. Initially, 1 × 10^4^ cancer cells were seeded into the upper invasion chambers without FBS. Then, DMEM containing 30% FBS was added to the lower chambers. After a 12 to 24-h culture, cells were fixed on the insert membranes using methanol and stained with a Crystal Violet Staining Solution (Solarbio, China). The invading cells were photographed and analyzed under a microscope at five different fields per well. To assess cell migration, the Transwell assay was performed once again using 24-well Corning Costar inserts with 8 μm pores that were not coated with Matrigel. Initially, 1 × 10^4^ cancer cells were planted into upper chambers, and DMEM containing 30% FBS was placed in the lower chambers. After culturing for 12 to 24 hours, the cells were fixed on the insert membranes using methanol and stained with a Crystal Violet Staining Solution (Solarbio, China). The migrated cells were photographed and assessed under a microscope at five fields per well.

### Multiplexed immunofluorescence staining protocol

Freshly cut pancreatic tumor tissue was subjected to deparaffinization and rehydration by incubating sections in Biodewax and Clear Solution for 10 minutes each, with three changes. The tissue was then dehydrated in pure ethanol for 5 minutes with three changes and washed in distilled water. To wash the sample, use a Rocker device and PBS (pH 7.4) three times for 5 minutes each. Choose the appropriate antigen retrieval buffer and heat extent based on the tissue characteristics. Circle the sample and block endogenous peroxidase. Then, wash the sample three times with PBS (pH 7.4) in a Rocker device for 5 minutes each and eliminate any excess liquid. Use a liquid blocker pen to mark the objective tissue. Immerse the sample in 3% H2O2 and incubate at room temperature for 15 minutes in a dark place. After washing the tissue samples three times with PBS (pH 7.4) in a Rocker device for 5 minutes each, block with serum by eliminating any excess liquid and marking the objective tissue with a liquid blocker pen. Cover the objective tissues with 10% donkey serum (if the primary antibody originated from goat) or 3% BSA (if the primary antibody originated from other sources) at room temperature for 30 minutes. For the first primary antibody, discard the blocking solution and incubate the slides overnight at 4C with E-cadherin (1:1000, Servicebio Technology Co., Ltd., Wuhan, China, No: GB14076)Place the slides in a wet box with a small amount of water and use the corresponding secondary antibody marked with HRP. Wash the slides three times with PBS (pH in a Rocker device) for 5 minutes each, and then discard the liquid.To cover the objective tissue with a secondary antibody, respond appropriately to the first primary antibody in the species and incubate at room temperature for 50 minutes in dark conditions. To prepare the CY3-TSA solution, wash the slides three times with PBS (pH 7.4) using a Rocker device for 5 minutes each time. Then, appropriately dilute the CY3-TSA solution (1:1000, Servicebio Technology Co., Ltd., Wuhan, China, No: G1223) with TBST. Incubate the slides with the diluted solution for 10 minutes in dark conditions. After incubation, wash the slides three times with TBST using a Rocker device for 5 minutes each time. Then, immerse the slides in EDTA antigen retrieval buffer (pH 8.0, Servicebio Technology Co., Ltd., Wuhan, China, No: G1206) for microwave treatment. Maintain the slides at a sub-boiling temperature for 8 minutes, followed by standing for 8 minutes and another sub-boiling temperature for 7 minutes. This process removes the primary antibodies and secondary antibodies combined with tissue. To prevent buffer solution from evaporating, incubate slides with the second primary antibody PD-L1 (1:3000, Servicebio Technology Co., Ltd., Wuhan, China, No: GB114196) diluted with PBS appropriately overnight at 4°C in a wet box containing a small amount of water. After incubation, wash the slides three times with PBS (pH 7.4) in a Rocker device for 5 minutes each. Then, remove the liquid slightly. To cover objective tissue with secondary antibody PD-L1, the second primary antibody in the species must be appropriately responded to. The incubation should be done at room temperature for 50 minutes in dark conditions. For the FITC-TSA solution, the slides should be washed three times with PBS (pH 7.4) in a Rocker device for 5 minutes each. The slides should then be incubated with FITC-TSA solution (1:3000, Servicebio technology co, LTD in Wuhan,China,No,G1222.) for 10 minutes in dark conditions. After washing the slides three times with TBST in a Rocker device for 5 minutes each, immerse them in EDTA antigen retrieval buffer (pH 8.0, Servicebio Technology Co., Ltd., Wuhan, China, No: G1206) and maintain at a sub-boiling temperature for 8 minutes. Stand for 8 minutes and then repeat the sub-boiling temperature for 7 minutes to remove the primary and secondary antibodies combined with tissue. To prevent buffer solution from evaporating, incubate the third primary antibody ITGA2 (1:200, bioss, Beijing, China, No: BSM-52613R) overnight at 4°C in a wet box containing a little water. Afterwards, the slides should be washed three times with PBS (pH 7.4) in a Rocker device for 5 minutes each. After the washing step, discard the liquid. Finally, incubate the third corresponding secondary antibody labeled with CY5 (diluted at 1:500, Servicebio Technology Co, LTD, Wuhan, China, No: G1232). Cover the objective tissue with the secondary antibody that appropriately responds to the second primary antibody in the species. Incubate the slides at room temperature for 50 minutes in a dark condition. DAPI (Servicebio technology co, LTD, Wuhan, China, No: G1012) is used as a counterstain for the nucleus. To apply DAPI staining, incubate the sample with DAPI solution at room temperature for 10 minutes in a dark place. Next, wash the slides three times with PBS (pH 7.4) using a Rocker device, with each wash lasting 5 minutes. After removing excess liquid, incubate the slides with a spontaneous fluorescence quenching reagent for 5 minutes, followed by a 10-minute wash under flowing water. Finally, mount the sample by washing it three times with PBS (pH 7.4) in a Rocker device, with each wash lasting 5 minutes. After washing, remove excess liquid and then coverslip the sample with an anti-fade mounting medium. The images were acquired and collected using a slice scanner. DAPI fluorescence was excited using a UV wavelength range of 330-380 nm, resulting in a blue emission at 420 nm. FITC fluorescence was excited using a wavelength range of 465-495 nm and emitted a green glow within the range of 515-555 nm. CY3 fluorescence was excited by a wavelength range of 510-560 nm, producing a red emission at 590 nm. In order to differentiate CY5 from CY3, which originally emitted red fluorescence, CY5 was modified to emit a pink glow. This modification involved adjusting the excitation wavelength to the range of 608-648 nm and the emission wavelength to the range of 672-712 nm.

### Immunofluorescence analysis

The tissue slices were placed on the PANNORAMIC panoramic slice scanner (3DHISTECH, Hungary) and moved gradually under the scanner’s lens. This allowed for imaging of the tissue slices, capturing all the tissue information, which was then scanned and imaged to create a folder containing all the tissue information. After opening the CaseViewer2.4 software from 3DHISTECH (Hungary), observations can be made at a magnification of 1-400 times. The Indica Labs-HighPlex FL v3.1.0 module in the Halo v3.0.311.314 analysis software was utilized for quantifying the number of positive and total cells in the target area, with the positive rate (%) being subsequently calculated.

### Immunohistochemical

Postoperative specimens were collected from pancreatic cancer patients and embedded in paraffin for routine analysis. Immunohistochemical methods were used, with serial sections made at a thickness of 4μm.After dehydration and hydration, the sections were repaired with sodium citrate antigen, blocked with goat serum, blocked with peroxide, added with the first antibody ITGA2 monoclonal antibody (1:400, bioss, Beijing, China, No : BSM-52613R); CD4 monoclonal antibody (1:50, bioss, Beijing, China, No:bsm-52469R); CD8 monoclonal antibody (1:1500, bioss, Beijing, China, No:bs-23447R) and the second antibody IGg polymer (1:200, bioss, Beijing, China, No: bs-0295G) under high temperature and high pressure environment, and finally stained with DAB, In this study, hematoxylin was used for blue reverse staining, followed by dehydration and fixation. Cells expressing ITGA2 were identified by brownish yellow staining in either the cell membrane or cytoplasm. For each section, we randomly selected five fields and averaged the results. The positive cells’ area was then divided into three grades: weak positive (+), if it accounted for ≤ 5%-25%; positive (++) if it accounted for ≥ 25%-50%; and strong positive (+++), if it accounted for more than 50% (19). Positive cells were identified by brown staining in the membrane or cytoplasm and were counted as CD4+T cells and CD8+T cells. The study measured the presence of CD4+ and CD8+ T cells in relation to tumor cells, with the results categorized as weakly positive (+) for ≤ 10/100 tumor cells, positive (++) for > 10-20/100 tumor cells, and strong positive (+++) for > 20/100 tumor cells, with three grades in total (20). The staining results were interpreted by three pathologists in a double-blind manner, and the section numbers were randomly determined, without any correspondence to the clinicopathological data.

### Statistical analysis

In this study, we used the x^2^ test to examine differences in categorical variables among groups of patients. For continuous variables, which had a non-normal distribution as determined by the Kolmogorov-Smirnov test, we employed the Wilcoxon rank-sum test to detect differences between groups of patients. To analyze correlations between continuous variables, we utilized Spearman’s rank correlations. The Kaplan-Meier method was used to estimate the overall survival (OS). OS was defined as the time interval from the start of treatment or surgery until the patient’s death from any cause or the last known date of being alive. Censoring of OS data was done at 5 years. To determine the difference in survival between groups, a log-rank test was performed. The OS data was analyzed using multivariate models of Cox regression. cells analysis of Statistical significance was assessed using the paired t-test, Student’s t-test, and one or two-way ANOVA, followed by Tukey’s multiple comparison tests. P value of 0.05 was considered significant for all analyses. Statistical analysis was performed using GraphPad Prism 8.0 software (GraphPad Software, San Diego, CA, USA) and JMP Pro 15 software (SAS Institute, Cary, NC, USA). Hypothesis testing was conducted at a two-sided significance level with α set to 0.05.

## Result

### The expression of ITGA2, PD-L1 and E-cad in pancreatic cancer samples was analyzed by multiplex immunofluorescence

**Figure 1:**
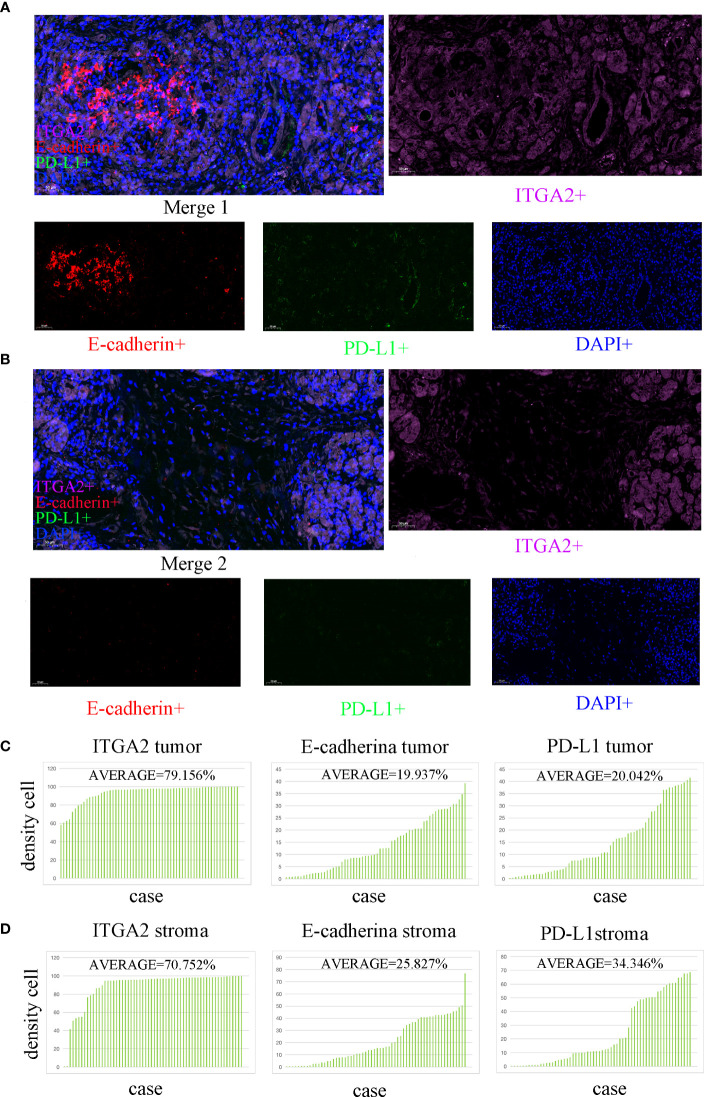
Multiple immunofluorescence staining was used to detect the expression of ITGA2, E-cadherin, and PD-L1 in both the tumor and stroma areas of pancreatic cancer tissue. (scale bar used was 50 µm). **(A)** The colocalization tumor map of ITGA2, E-cadherin, and PD-L1 expression was obtained along with DAPI staining.ITGA2-positive cells were represented in purple.Red color represents E-cadherin positive cells. Green represents PD- L1 positive cells; Blue represents DAPI staining. **(B)** This study analyzed the colocalization stroma map of ITGA2, E-cadherin, and PD-L1 expression with DAPI staining. **(C)** The average density of cells expressing ITGA2 in the tumor was 79. 156%, while E-cadherin and PD-L1 had densities of 19.973% and 20.042%,respectively. **(D)** In the stroma, the average density of cells expressing ITGA2 was 70.752%, while E-cadherin and PD-L1 had densities of 25.827% and 34.346%, respectively. Abbreviations used in the study include DAPI for 4,6-diamidino-2-phenylindole, PD-L1 for programmed cell death-Ligand 1, and ITGA2 for integrin subunit alpha2.

**Figure 2:**
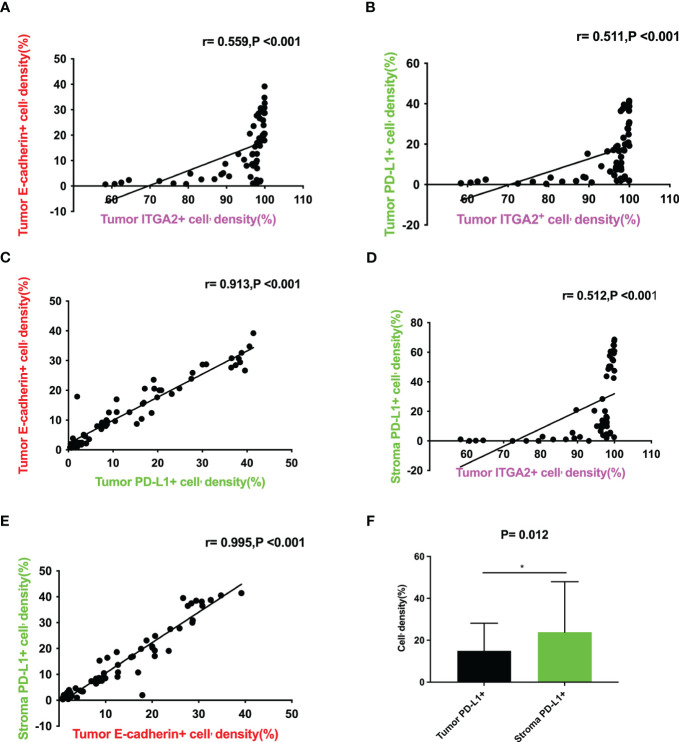
Correlation between the expression levels of ITGA2+ cell density, E-cadherin+ cell density in tumor and PD-L1+ cell density in tumor and stroma in pancreatic cancer tissues. **(A)**Scatter plot graph illustrating that tumor ITGA2+ cell’density(%) directly correlates with tumor E-cadherin+ cell’density(%) (r= 0.559,P<0.001); **(B)**Scatter plot graph illustrating that tumor ITGA2+ cell’density(%) directly correlates with tumor PD-L1+ cell’density(%) (r= 0.511,P<0.001); **(C)**Scatter plot graph illustrating that tumor PD-L1+ cell’density(%) directly correlates with tumor E-cadherin+ cell’density(%) (r= 0.913,P<0.001); **(D)**Scatter plotgraph illustrating that tumor ITGA2+ cell’density(%) directly correlates with stroma PD-L1+ cell’density(%) (r=0.512,P<0.001); **(E)** Scatter plot graph illustrating that tumor E-cadherin+ cell’density(%) directly correlates with stroma PD-L1+ cell’density(%) (r= 0.995,P<0.001); **(F)** Tumor ITGA2+ cell’density(%) VS stroma PD-L1+ cell’density(%) (P=0.012).

In the study analyzing 62 postoperative specimens of pancreatic cancer, it was found that, on average, the density of ITGA2+ cells was expressed in 79.156% of pancreatic cancer tissues. Similarly, the densities of E-cadherin+ and PD-L1+ cells were expressed in 19.973% and 20.042% respectively (**Figures 1A, C** **)**. In the stroma, the average expression of ITGA2+, E-cadherina+, and PD-L1+ cells density was 70.752%, 25.827%, and 34.346% respectively (**Figures 1B, D** **)**. Interestingly, the density of ITGA2+ cells density was not found to be correlated with age, sex, tumor marker CA125, or tumor size. In this study, ITGA2+ cells density were found to be significantly correlated with CA-199 (P= 0.004), CEA (P=0.03), TNM stage (P= 0.037), lymph node metastasis (P= 0.001) and local invasion (P= 0.02), indicating its importance in the diagnosis and growth of pancreatic cancer. On the other hand, E-cadherina+ cells density was not found to be correlated with age, gender, CEA, CA- 125, tumor size, and local invasion. However, it was significantly correlated with CA- 199 (P=0.013), TNM stage (0.023), and lymph node metastasis (P= 0.003).The density of PD-L1+ cells density that exhibit regulatory T cell immunity showed significant associations only with CA- 199 (P=0.014), CEA (P=0.032), and lymph node metastasis (P=0.009) in cases of pancreatic cancer, as shown in **Table 1**. To better understand the difference in PD-L1 cells density expression between the tumor and stroma areas in pancreatic cancer, we compared the density of positive cells in both regions. Our findings revealed that the expression of PD-L1 cells density in the stroma area was significantly higher than that in the tumor area (P=0.012, **Figure 2F**). This comparison is crucial for the treatment of pancreatic cancer. Our findings suggest indicate a significant correlation between ITGA2+ cells in the tumor area and E-cadherin+ (r=0.599, P< 0.001) and PD-L1+ (r =0.511, P <0.001) (**Figures 2A, B**). Meanwhile, E-cadherin+ cells are also significant with PD-L1+ (r =0.913, P <0.001) (**Figure 2C**) Additionally, we observed a positive correlation between ITGA2+ cells (r=0.512, P< 0.001) and E-cadherin+ (r=0.995, P<0.001) with PD-L1+ cells in the stromal region (**Figures 2D, E**), indicating a regulatory relationship among them.

**Table 1:** Relationship between ITGA2+ cells, E-cadherin+ cells, PD-L1+ cells and clinicopathological features.

		ITGA2+cell' density in tumor		E-cad+cell' density in tumor		PD-L1+cell' density in tumor	
	N	Mean±SD(%)	P Value	Mean±SD(%)	P Value	Mean±SD(%)	P Value
Age(year)			0.387		0.463		0.282
<60	35	94.49 ±7.95		12.87 ± 10.5		3.35 ± 12.37	
≤60	27	92.02 ± 12.87		14.94 ± 11.55		17.01 ± 14.09	
Gender			0.086		0.234		0.299
Male	44	95.91 ±6. 14		14.79 ± 1.09		16.06 ± 13.2	
Female	18	88. 18 ± 14.87		11.30 ± 1.00		12.21 ± 13.02	
Tumor markers							
CA19-9			0.0004		0.013		0.014
(+)	37	97. 1 ±0.55		16.51 ± 1.72		18.29 ±2. 13	
(-)	25	87.97 ±2.85		9.737 ± 1.97		10.02 ±2.41	
CA125			0.092		0. 1565		0.619
(+)	10	21.27 ±4.91		18. 19 ±3.93		94.93 ±2.50	
(-)	52	3.72 ± 1.72		12.93 ± 1.42		93. 13 ± 1.50	
CEA			0.03		0.055		0.032
(+)	25	96.87 ±0.86		16.94 ±2.07		19.26 ±2.65	
(-)	37	91.09 ±2.06		11.65 ± 1.72		12.04 ±2.04	
TNM			0.037		0.023		0.019
T1 +T2	26	89.76 ± 13.93		11.06 ± 1.05		17.79 ± 13.23	
T3 +T4	36	96.05 ±5.61		16.39 ± 1.01		17.23 ± 12.81	
Lymphatic metastasis			0.001		0.003		0.009
Yes	48	97.32 ±2.80		15.88 ± 1.03		17.28 ± 12.20	
No	14	80.03 ± 15.06		6.57 ± 12. 19		6.95 ± 13.64	
Tumor mass(cm)			0.311		0. 134		0.102
>2	35	95.27 ± 11.34		11.99 ± 1099		12.58 ± 12.45	
≦2	27	94.9 ±8.93		16.08 ± 10.03		18.01 ± 12.66	
Partial invasion			0.02		0. 113		0.098
Yes	42	97.26 ±2.75		15.25 ± 10.09		16.86 ± 12.54	
No	20	85.33 ± 15.06		10.68 ± 11. 15		10.93 ± 13.84	

### Expression of ITGA2, CD4 and CD8 in pancreatic cancer tissues and adjacent tissues

**Figure 3:**
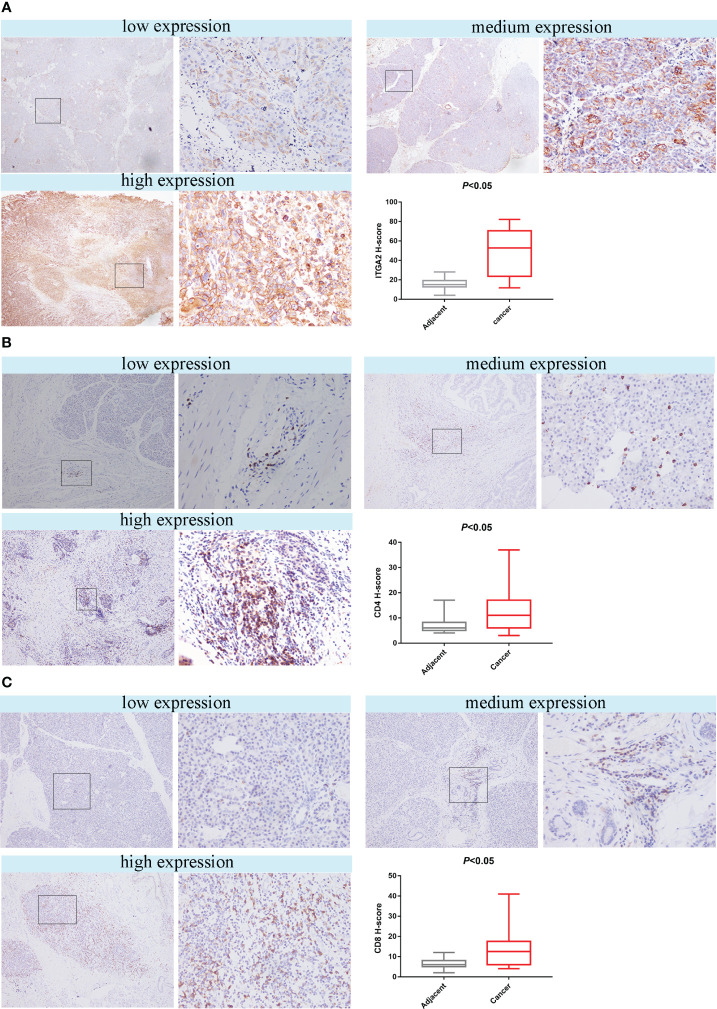
The study found that the expression of ITGA2, CD4 and CD8 in pancreatic cancer tissues and adjacent tissues were analyzed using HIC. The left scar bar represents 100× and the right scar bar represents 400×. **(A)** The results indicated that ITGA2 exhibited low, medium, and high expression levels in pancreatic cancer, and the H-score between the adjacent and cancerous tissues showed significant differences (P<0.05).In pancreatic cancer, the expression levels of CD4 **(B)** and CD8 **(C)** can be classified as low, medium, or high. The H-score indicates a significant difference (P < 0.05) between adjacent and cancer tissues for both CD4 and CD8.

**Figure 4:**
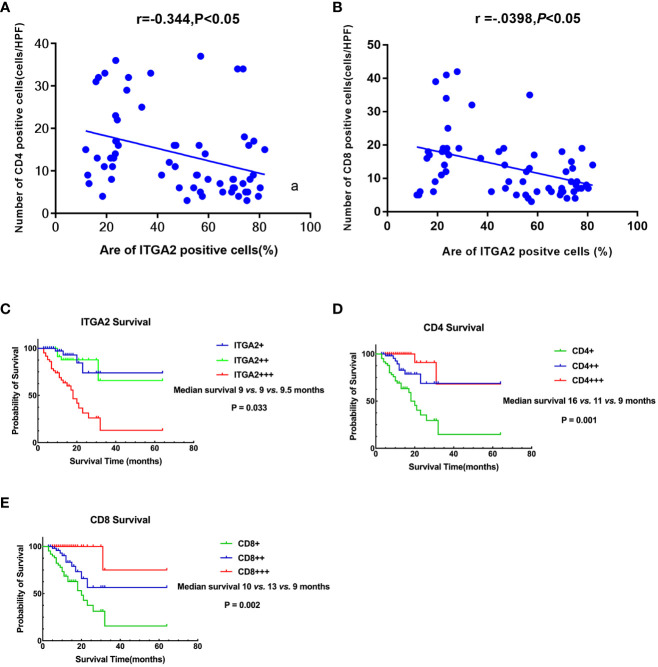
Correlation and Prognostic Signifificance between the expression levels of ITGA2, CD4,and CD8 in Pancreatic Cancer. **(A)** A scatter plot graph illustrates that the percentage of ITGA2 positive cells directly correlates with the number of CD4 positive cells (r= -0.344, P<0.05). **(B)** A scatter plot graph illustrates that the percentage of ITGA2 positive cells directly correlates with the number of CD8 positive cells (r= -0.398, P<0.05). **(C)** Patients with ITGA2+++ exhibited a shorter survival compared to patients with ITGA2++ and ITGA2+ (median survival: 9 vs. 9 vs. 9.5 months, P=0.033). **(D)** Patients with CD4+++ had a longer survival compared to patients with CD4++ and CD4+ (median survival: 16 vs. 11 vs. 9 months, P=0.001 ). (E) Patients with CD8+++ had a longer survival compared to patients with CD8++ and CD8+ (median survival: 10 vs. 13 vs. 9 months, P=0.002 ). + repersent weakly positive, ++ represent positive, +++ represent strong positive.

To investigate whether ITGA2 regulates immune function in pancreatic cancer, we conducted ITGA2, CD4, and CD8 assays on postoperative specimens from 62 patients with pancreatic cancer. The ITGA2 immunohistochemical positive reaction products were predominantly brown-yellow or tan, and were mainly distributed in the cell membrane and cytoplasm (**Figure 3A**). CD4 and CD8 were mainly distributed in the cytoplasm and cell membrane (**Figures 3B, C** **)**. In cancer tissues, the expression of ITGA2 was observed to have varying levels: weak expression was present in 25.8% (16/62) of cases, moderate intensity expression was found in 22.6% (14/62) of cases, and high expression was detected in 51.6% (32/62) of cases. Conversely, in adjacent tissues, the expression pattern of ITGA2 differed significantly. Weak expression was observed in 54.8% (34/62) of cases, while moderate intensity expression was only seen in 1.6% (1/62) of cases. Notably, no high intensity expression was observed in the adjacent tissues. The study found that in cancer tissues, the weak expression rate of CD4 was 46.8% (29 out of 60), while the moderate expression rate was 32.3% (20 out of 62), and the high expression rate was 21% (13 out of 62). In paracancerous tissues, the weak expression rate of CD4 was 38.75% (24 out of 62), while the moderate and high intensity expression rates were 0.In cancer tissues, CD8 expression was weak in 48.4% (30/62) of cases, moderate in 38.7% (24/62) of cases, and high in 12.9% (8/62) of cases. In adjacent tissues, the weak expression rate was 29% (18/62) and there were no cases of moderate or high intensity expression. The expression rates of ITGA2, CD4, and CD8 were found to be significantly higher in cancer tissues compared to adjacent tissues. Statistical analysis showed that the difference was significant (P < 0.05). Moreover, the protein expression level of ITGA2 in pancreatic cancer tissues was negatively correlated with the protein expression levels of CD4 and CD8 (r = -0.344, P < 0.05 and r = -0.398, P < 0.05, respectively).The study found that as the expression level of ITGA2 increased, the expression level of CD4 and CD8 protein decreased. This finding implies that ITGA2 may have a crucial role in the development of an immunosuppressive microenvironment within tumors, as depicted in **Figures 4A, B**.

### Correlation between the expression levels of ITGA2, CD4, CD8 and clinicopathological parameters in pancreatic cancer tissues

Pancreatic cancer exhibits high expression of ITGA2, however, the expression of CD4 and CD8 gradually decreases with tumor progression, rendering immunotherapy for advanced pancreatic cancer ineffective. In addition, we conducted further analysis on the expression of ITGA2, CD4, and CD8 in pancreatic cancer tissues, as well as their correlation with various clinicopathological parameters such as gender, age, tumor size, tumor location, histological grade, TNM stage, lymphatic metastasis, and local invasion. Our findings shed light on the potential significance of these markers in predicting clinical outcomes. The study findings indicate that the levels of ITGA2, CD4, and CD8 in tumor tissues are significantly associated with histological grade, TNM stage, lymph node metastasis, and local invasion (P < 0.05). However, there was no significant correlation observed with other clinicopathological parameters, as indicated in **Table 2**. In this study, univariate analysis of Cox model was conducted to determine the factors affecting the prognosis of patients with pancreatic cancer. The results revealed that histological grade, TNM stage, lymph node metastasis, local invasion, and the expression of ITGA2, CD4, and CD8 in tumor tissues were all associated with patient prognosis. However, upon conducting multivariate analysis, only local invasion was found to be an independent prognostic factor for overall survival time in patients with pancreatic cancer. These findings are presented in **Table 3**.

**Table 2:** The relationship between the expression of ITGA2, CD4 and CD8 in pancreatic cancer tissues and the clinicopathological features.

Clinical parameters	N	ITGA2			z	P	CD4			z	P	CD8			z	P
		+	++	+++			+	++	+++			+	++	+++		
gender					0.822	0.411				0.994	0.32				0.324	0.746
man	40	9	9	22			20	11	9			20	11	9		
women	22	7	5	10			10	9	3			10	9	3		
age(year)					0.341	0.733				1.951	0.061				1.913	0.056
>60	34	8	8	18			19	11	4			19	10	5		
≤60	28	8	6	14			10	9	8			11	10	6		
tumor size(cm)					1.321	0.186				0.322	0.747				0.265	0.791
>2	35	10	10	15			17	11	7			16	15	4		
≤2	27	6	4	17			12	9	6			14	9	4		
tumor location					0.544	0.586				0.041	0.968				0.745	0.456
head	40	12	9	19			19	15	6			18	17	5		
body and tail	22	4	8	13			10	5	7			11	7	4		
Differentiation					6.212	0.045				14.40	0.001				8.914	0.012
well	6	3	3	0			0	1	5			0	2	4		
moderate	45	9	10	26			22	16	7			25	16	4		
poor	11	4	1	6			7	3	1			6	3	2		
TNM stage					2.136	0.033				2.227	0.023				2.131	0.033
I-II	28	11	8	9			8	10	10			9	12	7		
III-IV	34	5	5	24			21	11	12			20	12	2		
lymph metastasis					2.058	0.041				2.972	0.003				1.961	0.05
Yes	40	5	7	28			19	9	12			19	12	9		
No	22	11	7	4			10	11	1			11	9	2		
Partial invasion					3.18	0.001				4.239	0.001				3.939	0.001
Yes	49	10	9	30			28	18	3			29	16	4		
No	13	6	5	1			1	2	10			2	1	10		

+ repersent weakly positive, ++ represent positive, +++ represent strong positive.

**Table 3:** Univariate and multivariate analysis of Cox model for clinical prognostic factors of pancreatic cancer (n = 62).

Clinical parameters	Univariate		Multivariate	
	HR(95%CI)	P	HR(95%CI)	P
Gender(Male vs Famale)	1.138(0.598-2.166)	0.694		
Age(years) (≤60 vs >60)	0.652 (0.352-1.208)	0.174		
Tumor size ( cm) (≤2, >2)	1.126(0.614-2.064)	0.702		
Tumor site (head vs body and tail)	0.766(0.398-1.487)	0.426		
Differentiation(well vs moderat vs Poor)	0.557(0.323-0.963)	0.036	1.495(0.639-3.495)	0.354
TNM staging(I-II vs III-IV)	2.249(1.166-4.337)	0.016	1.477 (0.704-3.097)	0.302
Lymph metastasis(Yes vs No)	0.524(0.276-0.966)	0.05	1.384 (0.646-2.968)	0.403
IITGA2 expression(+ vs ++ vs +++ )	1.521(1.003-2.306)	0.048	0.829 (0.489-1.404)	0.484
Partial invasion ( Yes vs No)	0.092(0.022-0.383)	0.001	0.092 (0.011-0.754)	0.0026
CD4 expression(+ vs ++ vs +++ )	0.472(0.303-0.735)	0.001	0.539(0.214-1.359)	0.19
CD8 expression(+ vs ++ vs +++ )	0.403 (0.233-0.696)	0.001	0.826(0.288-2.375)	0.723

### Prognostic significance of ITGA2, CD4 and CD8 in pancreatic cancer

This study evaluated the overall survival (OS) of pancreatic cancer patients by analyzing the expression of ITGA2, CD4, and CD8. The study successfully followed up with 62 patients with pancreatic cancer, achieving a success rate of 95.0%. The duration of the follow-up period ranged from 3 to 64 months, with an average survival time of 12.6 ± 10.1 months. The results of the study showed that patients with weak ITGA2 expression had a median survival of 9.5 months (range 4-15). Patients with moderate ITGA2 expression had a median survival of 9 months (range 9-20), while those with strong ITGA2 expression also had a median survival of 9 months (range 5-13). Additionally, patients with strong positive ITGA2 expression had a slightly longer median survival of 10 months (range 5- 15). The study found that patients who exhibited high levels of ITGA2 expression demonstrated a considerably lower 5-year survival rate compared to those who had either weak or moderate TIGA2 expression (P = 0.033, **Figure 4C**). Additionally, patients with weak CD4 expression exhibited a median survival time of 9 months (range: 5-12 months), while individuals with moderate CD4 expression had a median survival time of 11 months (range: 4-14 months). Furthermore, patients with strong CD4 expression displayed a median survival time of 16 months (range: 8-22 months).Based on the data presented in **Figure 4D**, it was observed that patients with strong CD4 expression exhibited a significantly higher 5-year survival rate when compared to individuals with weak and moderate CD4 expression (P = 0.001). Furthermore, the median survival time for patients with weak CD8 expression was found to be 9 months (range:5-14 months) , whereas those with moderate CD8 expression had a median survival time of 13 months(range:5-22 months). The study observed that patients exhibiting strong CD8 expression demonstrated a median survival time of 10 months(range:8-17 months). Furthermore, it was noted that patients with strong CD8 expression had a notably higher 5-year survival rate when compared to those showing weak or moderate CD8 expression (P = 0.002, **Figure 4E**).

### Silencing ITGA2 suppresses pancreatic cancer aggression ability *in vitro*

**Figure 5:**
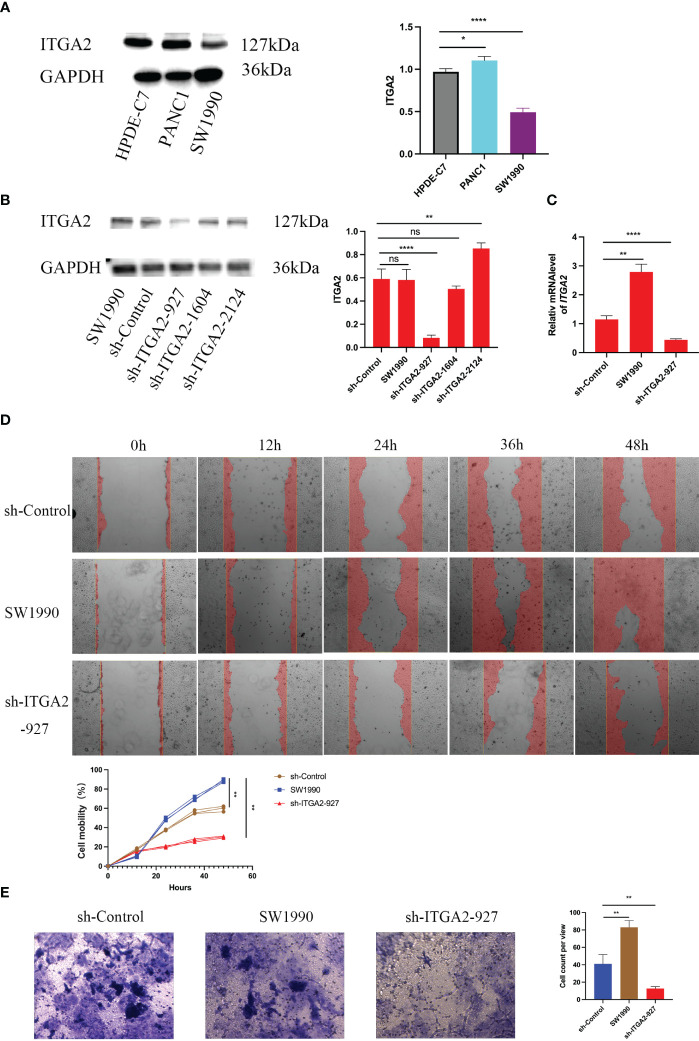
Silencing ITGA2 suppresses the aggressive ability of pancreatic cancer in vitro. Western blot **(A)**analysis of ITGA2 expression in HPDE-C7,PANC-1 and SW1990 cells .Western blot **(B)** and RT-PCR **(C)** analysis of ITGA2 expression in SW1990 cells infected with sh-Control or sh-ITGA2s.GAPDH served as an internal reference. Data presented as the mean ± SD of three independent experiments. Each sh-ITGA2 group was compared with the sh-Control group. Statistical analyses were performed using one-way ANOVA followed by Tukey’s multiple comparison tests. Statistical significance was denoted as *for P < 0.05, ** for P < 0.01 ,*** for P < 0.001 , **** for P < 0.0001 and ns respent no significance.SW1990 cells were infected with either sh-Control or sh-ITGA2-927. After forty-eight hours of culturing, the cells were harvested for migration assay **(D)** and cell invasion assay **(E)**. Representative images of migrated cells were shown based on a transwell assay. Each bar represents the mean ± SD of three independent experiments. Each sh-ITGA2 group was compared with the sh-Control group. Statistical analyses were performed using one-way ANOVA followed by Tukey’s multiple comparison tests. Statistical significance was denoted as ** for P < 0.01.

Since ITGA2 is highly expressed in pancreatic cancer tissues and is considered as a biomarker gene for poor prognosis of pancreatic cancer, we aimed to further investigate its biological role in pancreatic cancer. Initially, we examined the expression of ITGA2 in normal pancreatic ductal epithelial cells (HPDE-C7) and pancreatic cancer cell lines PANC-1 and SW1990. Our findings revealed that ITGA2 was expressed in all three cell lines; however, the expression of ITGA2 in SW1990 was slightly lower compared to the other two cell lines (**Figure 5A**). We choose SW1990 as the target silence cell. Following the knockdown of ITGA2 using short Harpine RNA (shRNA) in SW1990 cell lines (**Figures 5B, C**). Furthermore, the transwell assay revealed a significant decrease in migration and invasion abilities of cancer cells upon down-regulation of ITGA2 (**Figures 5D, E**). These findings suggest that knocking down ITGA2 could effectively inhibit pancreatic cancer proliferation and aggression in an *in vitro* setting.

### ITGA2 increases PD-L1 and EMT expression in pancreatic cancer cells

**Figure 6:**
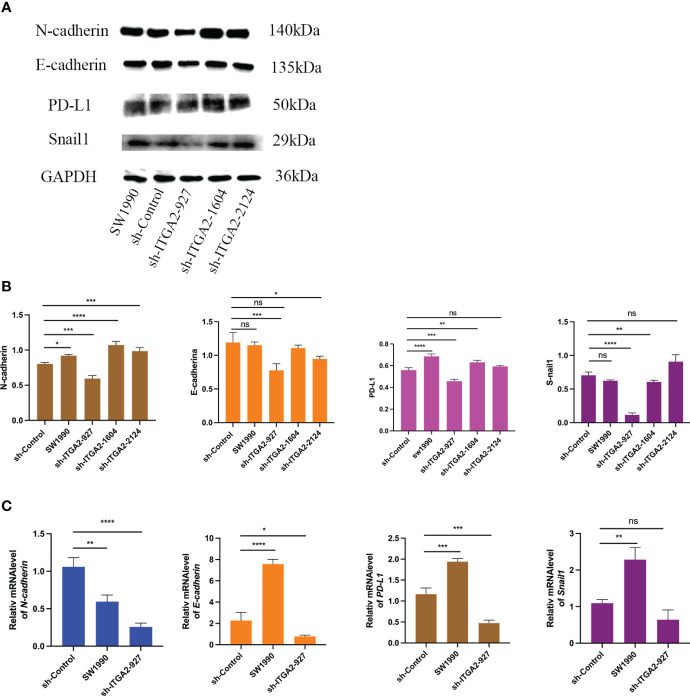
ITGA2 increases PD-L1 and EMT expression in pancreatic cancer cells. Forty-eight hours postinfection, SW1990 cells infected with sh-Control or sh-ITGA2s were harvested for Western blotting analysis **(A, B)** and RT-PCR analysis **(C)**. The data is presented as means ± SD (n = 3). Each sh-ITGA2 group was compared with sh-Control group. Statistical analyses were performed with one-way ANOVA followed by Tukey’s multiple comparison tests. *P < 0.05; **P < 0.01; ***P < 0.001; ****P<0.0001 and ns respent no significance.

Because ITGA2 is critical for promoting pancreatic cancer cell progression, we employed sh-ITGA2 to explore the relationship with EMT and PD-L. Our findings revealed a significant down-regulation of the E-cadherin, N-cadherin, Snail, and PD-L1 genes upon knockdown of ITGA2 using three independent sh-RNAs in SW1990 cells, particularly in the case of sh-ITGA2-927 (**Figure 6A**). Moreover, sh-ITGA2-927 exhibited a notable decrease in both mRNA and protein levels of E-cadherin, N-cadherin, Snail, and PD-L1 in SW1990 cells (**Figures 6B, C**). Based on these comprehensive results, it can be concluded that ITGA2 regulates the expression of EMT markers and PD-L1 in pancreatic cancer cells.

## Discussion

The development of tumors is influenced by various factors, including the tumor microenvironment. This environment promotes the continuous proliferation of tumor cells, the generation of new blood vessels, invasion and metastasis, and immune escape. Therefore, it is important to note that the generation of tumor cells is not solely attributed to a single factor, but rather is a complex process involving the interaction of multiple factors (19). The relationship between ITGA2 and cancer has attracted significant research attention in recent years. Recent studies have confirmed the association of the FAK/ATK signaling pathway with the epithelial-mesenchymal transition (EMT) in several malignant tumors, such as esophageal cancer, primary liver cancer, and pancreatic cancer (21). In esophageal malignant tumors, ITGA2 overexpression promotes tumor growth, invasion, and migration. However, by silencing or knocking out the ITGA2 gene, the FAK/AKT pathway can be inhibited, and the EMT phenotype can be downregulated (22). Pleckstrin homology like domain family A member 1 (PHLDA1) plays a crucial role in promoting the migration and proliferation of colon cancer cells by regulating the expression level of ITGA2 in colonic malignant tumors (23). Furthermore, ITGA2 has been found to be associated with the progression of acral melanoma and the clinical prognosis of patients suffering from the disease (24). ITGA2, a downstream effector molecule of the HMGA2-FOXL2 pathway, is implicated in early distant metastasis of tumor cells in gastric malignant tumors (25). While ITGA2 has been shown to promote cancer in a variety of tumors, its role in immune regulation within the tumor microenvironment of pancreatic cancer has yet to be confirmed.

The study included 62 patients who were diagnosed with resectable pancreatic cancer. Multiplexed Immunofluorescence was utilized to detect the expression levels of ITGA2, E-cadherin, and PD-L1. Considering the relationship between aging, invasion, and metastasis of pancreatic cancer, it is crucial to consider ITGA2 mutation as significant as CDKN2A, BRCA1/2, and PALB2 mutations in the diagnosis and treatment of pancreatic cancer. In our study, we examined the expression of EMT markers, specifically E-cadherin and PD-L1, in the same sample. Surprisingly, we found that both markers were expressed mainly in the stroma. Specifically, PD-L1 expression was not significantly high, with 20% in the tumor area and 34% in the stroma area. This expression level is lower than the 50% observed in lung cancer (26), which could explain the limited efficacy of current anti-PD- 1 immunotherapy in pancreatic cancer. Our analysis revealed a positive correlation between ITGA2, E-cad and PD-L1 in both the tumor and stroma. This reinforces the crucial role played by the integrin family in the regulation of metastasis and EMT plasticity in pancreatic cancer (8). Additionally, the overexpression of ITGA2 activates the STAT3 signaling pathway, leading to the up-regulation of PD-L1 expression and promoting malignant tumor invasion (14). ITGA2, E-cad and PD-L1 have been found to be associated with pancreatic cancer tumor markers CA19-9 and lymph node metastasis. CA19-9 is a specific marker used in the diagnosis of pancreatic cancer and is widely used in clinical practice. Additionally, if CEA + CA125 +/CA19-9> 1000 U/mL is present, it indicates a poor outcome for pancreatectomy in pancreatic cancer patients (27). The regulation of EMT by ITGA2 plays a crucial role in the growth of pancreatic cancer. Additionally, ITGA2 also regulates the expression of PD-L1 in pancreatic cancer, which leads to changes in the immune microenvironment and further promotes the progression and metastasis of the cancer. Therefore, understanding the role of ITGA2 in these processes is of great significance in the development of effective treatments for pancreatic cancer.

To investigate the effect of ITGA2 on the immune microenvironment of pancreatic cancer, we conducted immunohistochemistry to detect the expression of ITGA2, CD4+T cells, and CD8+T cells in tumor tissues. We also analyzed the relationship between these markers and the clinicopathological characteristics and prognosis of the patients. Our study revealed a significant correlation between

ITGA2 and histological grade, TNM stage, lymph node metastasis, and local invasion (P < 0.05). These findings suggest that ITGA2 may have a pro-tumor effect in the development and occurrence of pancreatic tumors. Regulatory T cells (Treg cells) are a subset of CD4+ T cells that possess negative regulatory functions. They are capable of secreting various immunosuppressive factors, such as TGF-β, IL- 10, and CCL5, within tumor cells, thereby contributing to the formation of the tumor immune microenvironment (28). Research has demonstrated that regulatory T (Treg) cells possess the capability to impede the functioning of CD4+T and CD8+T cells. A significant abundance of Treg cells can be observed in both tumor tissues and peripheral blood of patients with pancreatic cancer, and this presence is associated with an unfavorable prognosis (29). Additionally, CD4+T cells and CD8+T cells play a crucial role in the anti-tumor response in various cancers, including colorectal cancer and breast cancer (30). Within the tumor immune microenvironment, the quantity of CD4+T and CD8+T cells gradually diminishes as the tumor progresses, resulting in an imbalance in immune function and eventual evasion of immune surveillance (31). Furthermore, recent research has indicated that interleukin-35 (IL-35) is highly expressed in pancreatic cancer tissues and is closely associated with the prognosis of pancreatic cancer patients (32). Treg cells secrete IL-35 which can activate GP130-STAT1 signaling pathway, leading to overexpression of ICAM1 and secretion of CCL5. This further mediates the recruitment of monocytes and macrophages into tumor tissues, promoting the generation and metastasis of tumor neovas (33). Our study discovered that the expression of CD4+ and CD8+ T cells in pancreatic cancer tissues was significantly reduced, while the expression of ITGA2 was increased. This indicates that ITGA2 not only contributes to the development of pancreatic cancer, but also has an immunosuppressive effect in the tumor immune microenvironment, which further promotes the progression of pancreatic cancer. Further research is needed to explore the connection between ITGA2 and Treg cells in promoting the formation of an immunosuppressive microenvironment, as well as their role in tumor development. Additionally, the underlying mechanisms of this relationship should also be investigated. The study included clinicopathological data of pancreatic cancer and the expression of ITGA2, CD4+T and CD8+T cells in tumor tissues for Cox model analysis. Results from the univariate analysis revealed that the expression of ITGA2+ cells was significantly associated with overall survival time, as well as the expression of CD4+ and CD8+T cells, degree of tissue differentiation, TNM stage, lymphatic metastasis, and local invasion in pancreatic cancer. The multivariate analysis results indicated that local invasion was the only independent prognostic factor for overall survival in pancreatic cancer. Although the expression level of ITGA2+ cells in pancreatic tumor tissues was not found to be an independent prognostic factor for patients, it is still considered a potential prognostic indicator for patients.

The results obtained in pancreatic cancer patients indicated high expression of ITGA2, E-cadherin, and PD-L1 in both the tumor and stroma. In order to further comprehend the regulatory relationship between ITGA2, EMT, and PD-L1 in pancreatic cancer cells, we conducted a detection study. Although previous studies have demonstrated the significant roles of PD-L1 and EMT in pancreatic cancer (34–37), it remains unclear whether ITGA2 can regulate these factors. At first, we demonstrated that ITGA2 high expressed in several pancreatic cancer cells and knocking down ITGA2 inhibited pancreatic cancer cell proliferation and invasion suggesting that ITGA2 might play an essential role in the pathogenesis of PDAC. Additionally, our findings point to ITGA2 acting as an oncogenic protein to promote the progression of pancreatic cancer. Furthermore, our data showed that E-cadherina, N-cadherina, Snail and PD-L1 expression were selectively down-regulated after knocking down ITGA2 in SW1990 cells. These results suggest that ITGA2 might be critical in modulating the anti-tumor efficiency of immune checkpoint-based therapy and the tumor growth process.

In our study, we observed the expression of ITGA2, E-cad, and PD-L1 in both tumor tissues and stroma of patients with pancreatic cancer. We found a positive correlation between ITGA2 and E-cadherina as well as PD-L1 and ITGA2 can regulate MET and PD-L1 in pancreatic cells. Furthermore, we observed significant correlations between these markers and CA- 199 levels and lymph node metastasis. In our study, we examined the expression profile of ITGA2 in pancreatic cancer and found that it was present in the majority of patients, particularly in those with moderately and poorly differentiated tumors. Moreover, we observed that the significance of ITGA2 in pancreatic cancer was influenced by factors such as lymph node status, degree of differentiation, and local invasion status. Further experimental verification is needed to confirm the immunosuppressive role of ITGA2 in the immune regulation of pancreatic cancer microenvironment, as there is a negative correlation between ITGA2 and CD4 and CD8.

## Conclusion

ITGA2 can serve as a novel target for both the development and treatment of pancreatic cancer. Its high expression has been found to impact the MET status and the expression of PD-L1, CD4, and CD8 in the immune microenvironment of pancreatic cancer patients, leading to a poor prognosis. Therefore, targeting ITGA2 is a promising approach to improve the effectiveness of checkpoint immunotherapy and inhibit tumor growth in pancreatic cancer.

## Data availability statement

The original contributions presented in the study are included in the article/**Supplementary Material**, further inquiries can be directed to the corresponding authors.

## Ethics statement

The Biomedical Research Ethics Committee of the School of Medicine at Dali University approved the present study [approval number: SYXK(yunnan)2018-0002]. Written informed consent was obtained from the individual(s) for the publication of any potentially identifiable images or data included in this article.

## Author contributions

LJ initiated the research and drafted the manuscript; YT and YC designed the project and performed the statistical analysis. YD and XL completed all experiments; LJ,YT and YC confirm the authenticity of all the raw data. YT,HS,YC,ZS completed Surgical procedures and specimen collection. ZQL, XL, ZL, and BD analyzed the data and assisted in the experiments; YT provided practical technical guidance and analyzed the data; and JH checked the language issues and assisted in the experiments. All authors read and approved the final manuscript.

## Supplementary material

Supplementary Figure 1 Western blot test for pancreatic cells. **(A)** Western blot analysis of ITGA2 expression in HPDE-C7,PANC-1 and SW1990 cells; **(B)** Western blot analysis of ITGA2 expression in SW1990 cells infected with sh-Control or sh-ITGA2s(sh-ITGA2-927,sh-ITGA2-1604 and sh-ITGA2-2124); **(C)** Western blot analysis of Snail1,N-cadherin,E-cadherin and PD-L1 expression in SW1990 cells infected with sh-Control or sh-ITGA2s (sh-ITGA2-927,sh-ITGA2-1604 and sh-ITGA2-2124).
