# Supplementary material for: High expression ITGA2 affects the expression of MET, PD-L1, CD4 and CD8 with the immune microenvironment in pancreatic cancer patients
Source: Front Immunol. 2023 Oct 10;14:1209367. doi: 10.3389/fimmu.2023.1209367 (PMC10594995; doi:10.3389/fimmu.2023.1209367)
Supplement: Supplementary file 1 [file Table_1.docx]

**Supplementary Table1|Baseline characteristics of patients divided into level of physical activity**

| **Variables** | **Number** | **%** |
| --- | --- | --- |
| Age((years) |  |  |
| > 60 | 34 | 54% |
| ≤ 60 | 28 | 46% |
| Gender |  |  |
| Male | 40 | 64% |
| Female | 22 | 36% |
| Tumor markers |  |  |
| CA19-9(ku/L) |  |  |
| >37 (+) | 37 | 59% |
| ≤37 (-) | 25 | 41% |
| CA125(ku/L) |  |  |
| >35 (+) | 10 | 16% |
| ≤35 (-) | 52 | 84% |
| CEA(µg/L) |  |  |
| >5 (+) | 25 | 40% |
| ≤5(-) | 37 | 60% |
| TNM |  |  |
| Ⅰ-Ⅱ | 15 | 24% |
| Ⅲ-Ⅵ | 47 | 76% |
| Lymphatic metastasis |  |  |
| Yes | 40 | 64% |
| No | 22 | 36% |
| Tumor mass(cm) |  |  |
| >2 | 35 | 56% |
| ≦2 | 27 | 44% |
| Pathology |  |  |
| High | 6 | 10% |
| Middile | 45 | 73% |
| Low | 11 | 17% |
| Partial invasion |  |  |
| Yes | 43 | 69% |
| No | 19 | 31% |
| tumor location |  |  |
| head | 42 | 67% |
| body and tail | 20 | 33% |
