# Supplementary material for: High expression ITGA2 affects the expression of MET, PD-L1, CD4 and CD8 with the immune microenvironment in pancreatic cancer patients
Source: Front Immunol. 2023 Oct 10;14:1209367. doi: 10.3389/fimmu.2023.1209367 (PMC10594995; doi:10.3389/fimmu.2023.1209367)
Supplement: Supplementary file 2 [file Table_2.docx]

**Supplementary Table2|Sequences of gene-specific sh-RNAs**

| ITGA2  (human)  shRNA-2124 | /rC//rA//rA//rC//rA//rG//rA//rA//rU//rG//rC//rU//rC//rA//rG//rA//rU//rA/TT |
| --- | --- |
|  | /rU//rA//rU//rC//rG//rA//rG//rC//rA//rU//rU//rC//rU//rU//rG//rU//rU//rG/TT |
| ITGA2  (human)  shRNA-927 | /rG//rC//rG//rA//rC//rG//rA//rA//rG//rU//rG//rC//rU//rA//rC//rG//rA//rA//rA/TT |
|  | /rU//rU//rU//rC//rG//rU//rA//rG//rC//rA//rC//rU//rU//rC//rG//rU//rC//rG//rC/TT |
| ITGA2  (human)  shRNA-1604 | /rG//rU//rU//rC//rA//rG//rU//rU//rG//rA//rU//rG//rU//rG//rG//rA//rU//rA//rA/TT |
|  | /rU//rU//rA//rU//rC//rC//rA//rC//rA//rU//rC//rA//rA//rC//rU//rG//rA//rA//rC/TT |
| sh-Control | purchased from Sigma-Aldrich |
