# Supplementary figures and images for: High expression ITGA2 affects the expression of MET, PD-L1, CD4 and CD8 with the immune microenvironment in pancreatic cancer patients
Source: Front Immunol. 2023 Oct 10;14:1209367. doi: 10.3389/fimmu.2023.1209367 (PMC10594995; doi:10.3389/fimmu.2023.1209367)

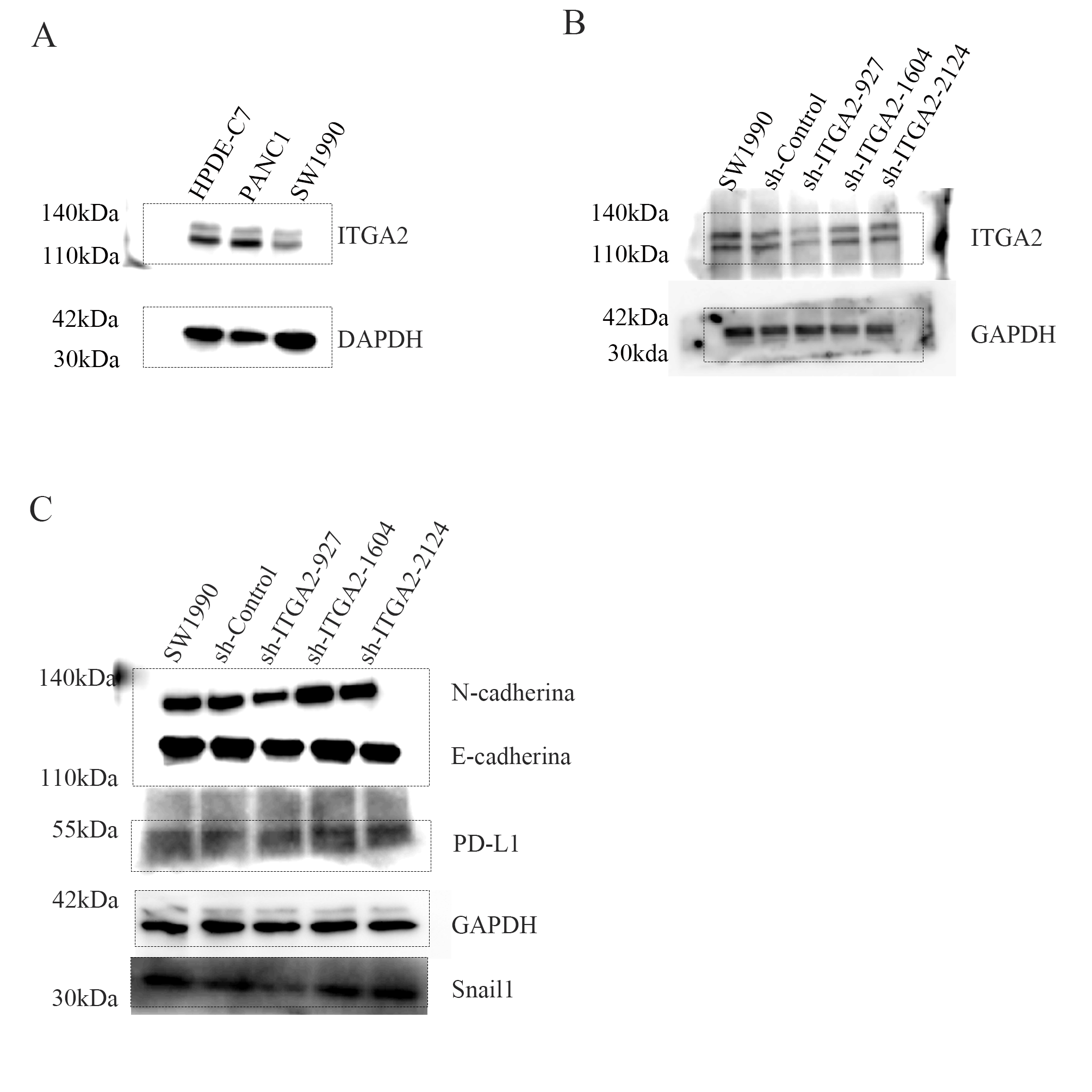

Supplement: Supplementary Figure 1 — Western blot test for pancreatic cells. (A) Western blot analysis of ITGA2 expression in HPDE-C7,PANC-1 and SW1990 cells; (B) Western blot analysis of ITGA2 expression in SW1990 cells infected with sh-Control or sh-ITGA2s(sh-ITGA2-927,sh-ITGA2-1604 and sh-ITGA2-2124); (C) Western blot analysis of Snail1,N-cadherin,E-cadherin and PD-L1 expression in SW1990 cells infected with sh-Control or sh-ITGA2s (sh-ITGA2-927,sh-ITGA2-1604 and sh-ITGA2-2124). [file Image_1.tif]
